# Ubiquitination of alpha-synuclein filaments by Nedd4 ligases

**DOI:** 10.1371/journal.pone.0200763

**Published:** 2018-07-18

**Authors:** Thomas Mund, Masami Masuda-Suzukake, Michel Goedert, Hugh R. Pelham

**Affiliations:** MRC Laboratory of Molecular Biology, Cambridge, United Kingdom; George Washington University, UNITED STATES

## Abstract

Alpha-synuclein can form beta-sheet filaments, the accumulation of which plays a key role in the development of Parkinson’s disease, dementia with Lewy bodies and multiple system atrophy. It has previously been shown that alpha-synuclein is a substrate for the HECT domain-containing ubiquitin ligase Nedd4, and is subject to ubiquitin-mediated endosomal degradation. We show here that alpha-synuclein filaments are much better substrates for ubiquitination *in vitro* than monomeric alpha-synuclein, and that this increased susceptibility cannot be mimicked by the mere clustering of monomers. Recognition by Nedd4 family enzymes is not through the conventional binding of PPxY-containing sequences to WW domains of the ligase, but it also involves C2 and HECT domains. The disease-causing alpha-synuclein mutant A53T is a much less efficient substrate for Nedd4 ligases than the wild-type protein. We suggest that preferential recognition, ubiquitination and degradation of beta-sheet-containing filaments may help to limit toxicity, and that A53T alpha-synuclein may be more toxic, at least in part because it avoids this fate.

## Introduction

The protein alpha-synuclein plays a major role in Parkinson’s disease, dementia with Lewy bodies and multiple system atrophy. In these diseases, brain cells accumulate aggregates of altered alpha-synuclein that has formed beta-sheet filaments, and this transformation into beta-sheet polymers is thought to underlie cellular toxicity and hence disease [1].

Abnormal proteins are typically substrates for quality control processes, including ubiquitin-mediated degradation by the proteasome, or by autophagy, and some aggregated alpha-synuclein is known to be ubiquitinated [2, 3]. Previous studies have shown that alpha-synuclein can be ubiquitinated by Nedd4, a member of the HECT-WW family of ubiquitin ligases, and that Nedd4 may serve to limit the concentration of alpha-synuclein and hence the extent of cellular toxicity [4]. Indeed, overexpression of Nedd4 can increase endosomal degradation of alpha-synuclein and protect cell viability [5, 6].

Nedd4 ubiquitin ligases often recognise substrates by interaction of their WW domains with motifs of the form PPxY (PY motifs) [7–10]. Alpha-synuclein does not contain a canonical PY sequence, but has proline-rich stretches near its C terminus, and it has been suggested that these stretches may underlie recognition by Nedd4 ligases [4, 6]. A complication is that the state of the protein that is ubiquitinated–monomeric versus filamentous–has not been determined, and thus attempts to map recognition sequences by deletion can potentially be complicated by changes in the propensity for alpha-synuclein to form filaments, or in other properties such as membrane binding.

Here we show that filamentous alpha-synuclein is a very much better *in vitro* substrate for ubiquitination than the monomeric protein. Recognition of filaments is not mediated by PY sequences, and is not simply a consequence of clustering monomers, but is likely to be a consequence of the conformational change that accompanies beta-sheet formation. The A53T mutant that leads to early-onset Parkinson’s disease [11] is ubiquitinated much less efficiently, despite forming filaments. This raises the possibility that specific ubiquitination of newly-formed filaments might help to limit cell toxicity.

## Results

### *In vitro* ubiquitination of alpha-synuclein

**Fig 1:**
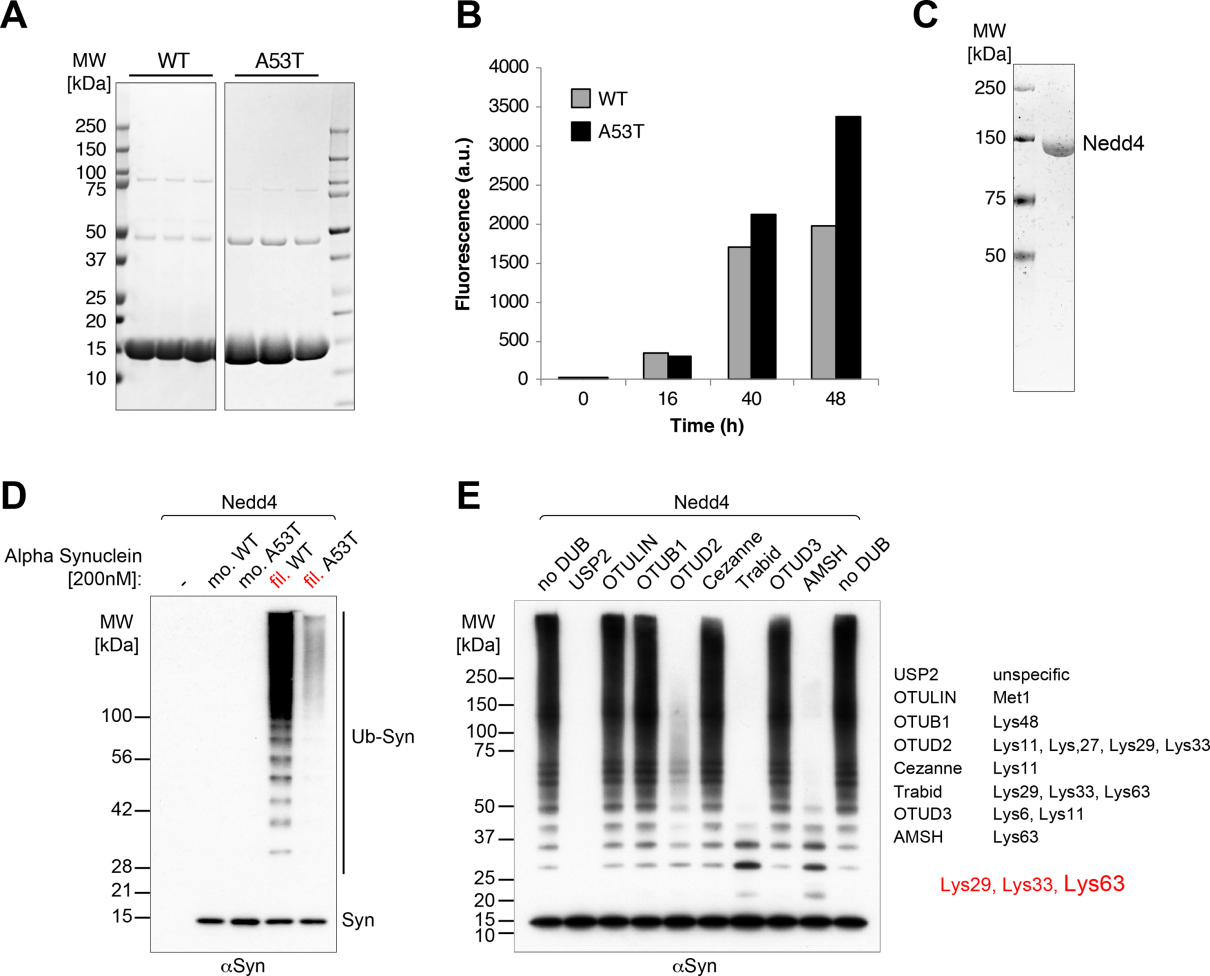
Efficient *in vitro* ubiquitination of alpha-synuclein filaments by Nedd4. Ubiquitination assays of Nedd4 with alpha-synuclein (antibody Syn-1). (A) Coomassie-stained gels of purified wild-type (WT) and A53T mutant alpha-synuclein. (B) Time course of polymerization of WT and mutant alpha-synuclein assayed by thioflavin dye fluorescence (see Methods). (C) Coomassie-stained gel of purified Nedd4 ligase. (D) *In vitro* ubiquitination assays using Nedd4 [200nM] with alpha-synuclein [200nM] showing efficient ubiquitination of filamentous (fil.) wild-type alpha-synuclein with strongly reduced modification of the filamentous A53T mutant and hardly any detectable ubiquitination of the monomeric forms (mo.). (E) Ubiquitination assays of Nedd4 [150nM] with alpha-synuclein filaments [600nM] in the presence of ubiquitin chain-type specific deubiquitinases as indicated [UbiCREST, [14]] demonstrating mainly K63, but also some K29 and K33, linkages.

We prepared recombinant alpha-synuclein (Fig 1A), both in monomeric form, and in the form of filaments produced by shaking, as described previously [12] (Fig 1B). Equal amounts of protein were then added to an *in vitro* ubiquitination assay containing purified Nedd4 ligase (Fig 1C). Fig 1D shows that the monomeric protein was largely unaltered, but that the filaments were excellent substrates for polyubiquitination. A previous report described ubiquitination of presumably monomeric alpha-synuclein by Nedd4 [4]. Our findings are not necessarily in conflict with this, as we did observe weak activity on monomers. Here, we focus on the filament-specific activity. Strikingly, filaments of the A53T mutant of alpha-synuclein, which polymerises at least as efficiently as the wild-type protein [13] (Fig 1B), were a much poorer substrate for ubiquitination than wild-type filaments (Fig 1D).

To characterise the polyubiquitin chains that were produced, we incubated them with a series of deubiquitinases of varying specificities, as shown in Fig 1E. The chains were largely abolished by incubation with AMSH, which is specific for K63 linkages, but they also showed sensitivity to OTUD2, which has other specificities. From the pattern of digestion, we conclude that the chains include mostly K63 linkages, but also some K29 and/or K33 junctions. These results are consistent with previous measures of Nedd4-induced ubiquitin chain specificity on alpha-synuclein, determined using mutant ubiquitin species [4, 6].

**Fig 2:**
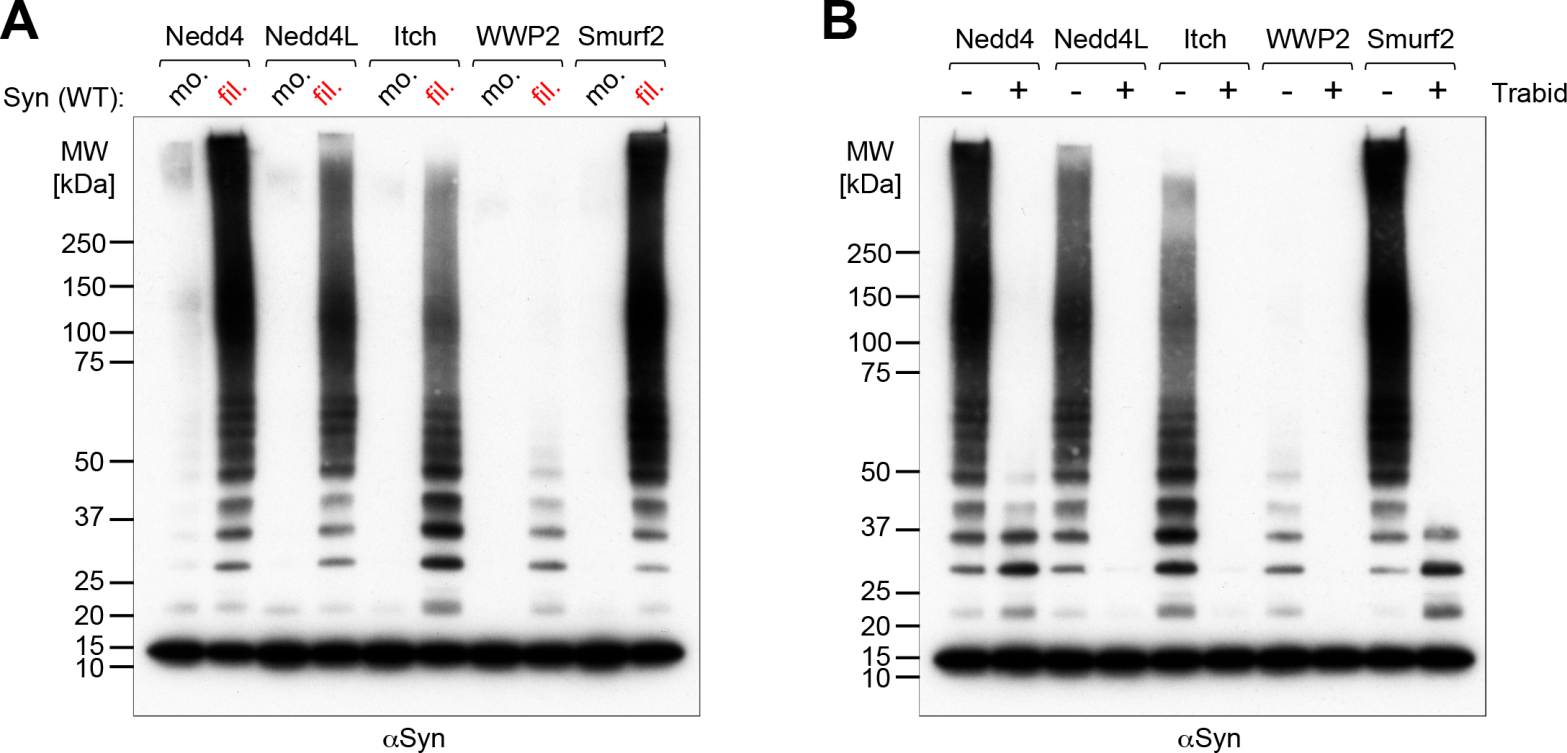
*In vitro* ubiquitination of alpha-synuclein by different Nedd4-type ligases. Western blots of *in vitro* ubiquitination assays using different members of the Nedd4 family [250nM] with alpha-synuclein [600nM] (antibody Syn-1). (A) Comparing the ability of the Nedd4-type E3 ligases to ubiquitinate monomeric (mo.) or filamentous (fil.) alpha-synuclein indicates a general preference for filaments. (B) Ubiquitination assays of different Nedd4 family members with filamentous alpha-synuclein with or without the ubiquitin chain-type specific deubiquitinase Trabid as indicated above the panel. In each case the ubiquitin chains were sensitive to Trabid, implying similar linkages.

We next compared the activity of Nedd4 with that of some other members of the Nedd4 family, to see whether filament specificity was a general phenomenon. Fig 2A shows that although Nedd4 had high activity, in agreement with previous findings [4], alpha-synuclein filaments were also substrates for Nedd4L, Itch and Smurf2, though only a weak substrate for WWP2. In each case the ubiquitin chains were sensitive to Trabid, implying linkages similar to those made by Nedd4 (Fig 2B).

### Ligase domains required for recognition of alpha-synuclein

The Nedd4 family of ubiquitin ligases is, to varying extents, autoinhibited by intramolecular interactions with the catalytic HECT domain. The nature of this interaction varies between different family members. With Nedd4 and Nedd4L little inhibition is apparent *in vitro*, for Smurf2 inhibition is mediated by the C2 domain, and for Itch sequences in and around the WW domains are important, whereas the C2 domain is not [15–19]. For a protein to be an efficient substrate for purified enzyme, it must be capable, not only of interacting with the ligase, but also of doing so in a manner which leads to its activation.

**Fig 3:**
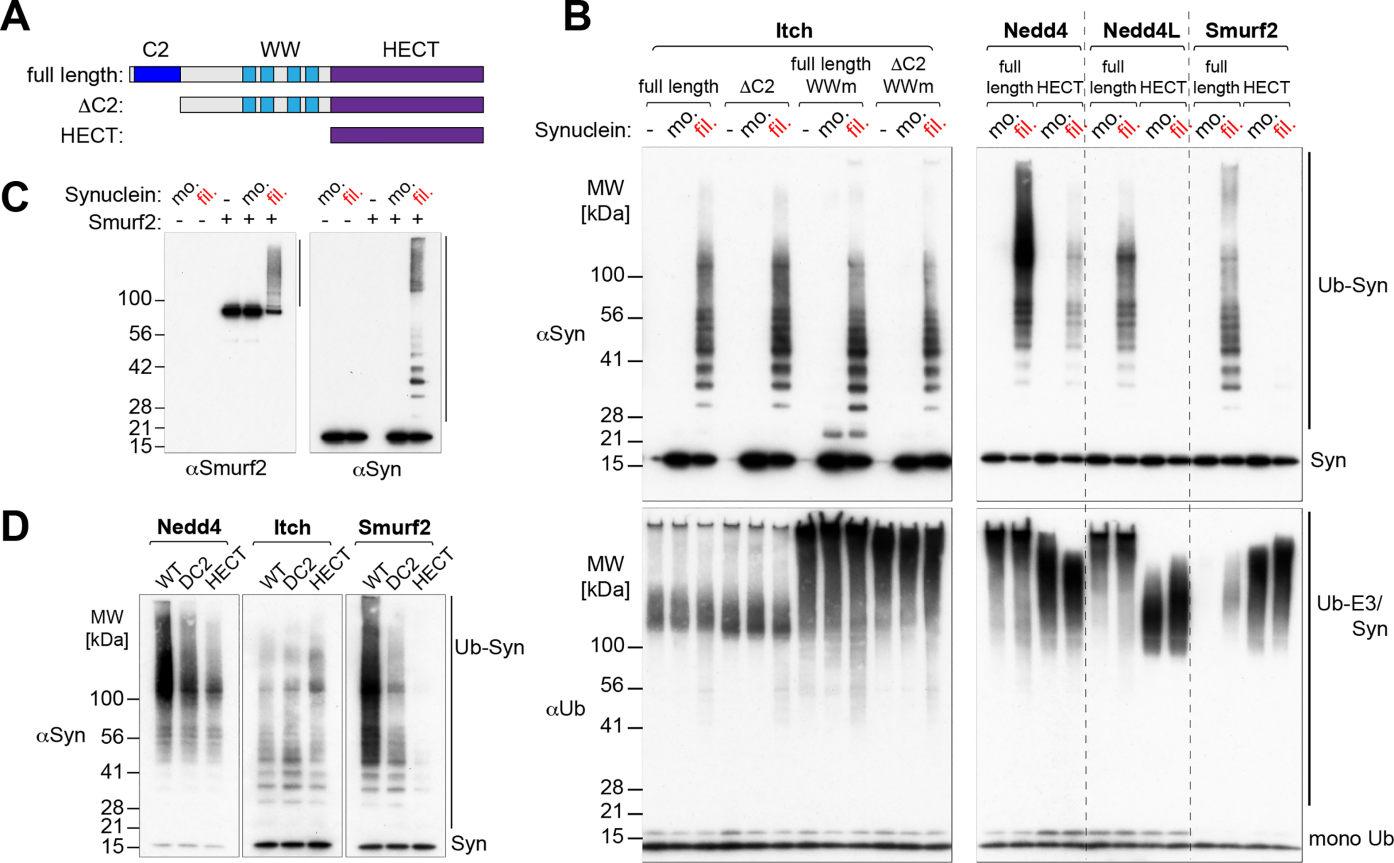
Ligase-specific recognition of alpha-synuclein filaments. (A) Cartoon of the modular architecture of Nedd4-type E3 ligases and overview of the constructs used in this study. (B) Western blots of *in vitro* ubiquitination assays comparing different Itch versions (full-length, ΔC2, WW mutants [175nM, left panel]) and full-length or HECT-only versions of Nedd4, Nedd4L and Smurf2 [100nM, right panel] in their ability to ubiquitinate alpha-synuclein [600nM, upper panels]. The lower panels (anti-ubiquitin blot) show overall ubiquitination, demonstrating the activity of the E3 ligase constructs by their ability to auto-ubiquitinate. Note that full-length Smurf2 is auto-inhibited in the presence of monomeric alpha-synuclein. (C) Activation of autoinhibited Smurf2 by alpha-synuclein filaments. (D) Western blots of *in vitro* ubiquitination assays comparing full-length, ΔC2 or HECT-only versions of Nedd4, Itch and Smurf2 [100nM] in their ability to ubiquitinate alpha-synuclein [400nM], indicating ligase-specific recognition of filaments (antibody Syn-1). Three sections of the same exposure of the same gel are shown.

As an example, we analysed mutants of Itch in which either the C2 domain had been deleted, or each WW domain had been mutated, or both changes had been introduced (Fig 3A). Though mutation of the WW domains stimulated enzyme activity, as measured by auto-ubiquitination, neither this, nor deletion of the C2 domain affected the activity on filamentous alpha-synuclein (Fig 3B). The simplest explanation is that direct interaction of the HECT domain with the filaments is sufficient to activate ubiquitination.

Different ligases gave different results. Data for Nedd4 shows that its isolated HECT domain had activity on the filaments, consistent with the Itch data. However, activity of the full-length ligase was substantially higher, indicating that there must also be additional interactions (Fig 3B). In contrast, isolated Nedd4L and Smurf2 HECT domains failed to modify the filaments, though they had robust autoubiquitination activity, implying that for these enzymes interactions are located exclusively outside the HECT domain (Fig 3B).

Unlike Nedd4, purified Smurf2 is efficiently autoinhibited, a feature that depends on the C2 domain, yet it also efficiently ubiquitinated alpha-synuclein filaments. This suggests that the filaments can activate the enzyme, which we confirmed by examining the modification of Smurf2 itself and of alpha-synuclein using specific antibodies (Fig 3C). Filaments indeed induced Smurf2 autoubiquitination. A likely explanation is that they interacted with the C2 domain, displacing it from its inhibitory interaction with the HECT domain. Fig 3D compares the activity of full-length Smurf2 with a C2 deleted version and with the isolated HECT domain alone. It can be seen that removal of the C2 domain significantly reduced ubiquitination of filaments, consistent with this playing a major role in their recognition. Nedd4 showed a similar dependence on the C2 domain (in addition to the HECT domain), whereas Itch did not, in agreement with earlier results. Strikingly, with all three enzymes, there seemed to be at most a modest contribution from the WW domains, which lie between the C2 and HECT domains. Since WW domains are the binding site for PY elements, we conclude that recognition of alpha-synuclein filaments is not primarily driven by PY-WW binding, but rather involves filament-specific interactions that require the C2 and/or HECT domains.

### Clustering of alpha-synuclein molecules does not result in efficient ubiquitination

We have recently shown that activation of Nedd4 ligases by PY-containing sequences is greatly enhanced if the PY motifs are clustered in large arrays, with the presence of these arrays helping to keep the ligase in an active conformation [20]. The formation of alpha-synuclein filaments also has the effect of clustering many monomers together, raising the question of whether it is this clustering alone that makes them good ubiquitination substrates, by providing a dense array of binding sites that are present in the monomer, or whether the conformational change involved in the formation of beta-sheet structures generates qualitatively different binding sites for the ligase.

**Fig 4:**
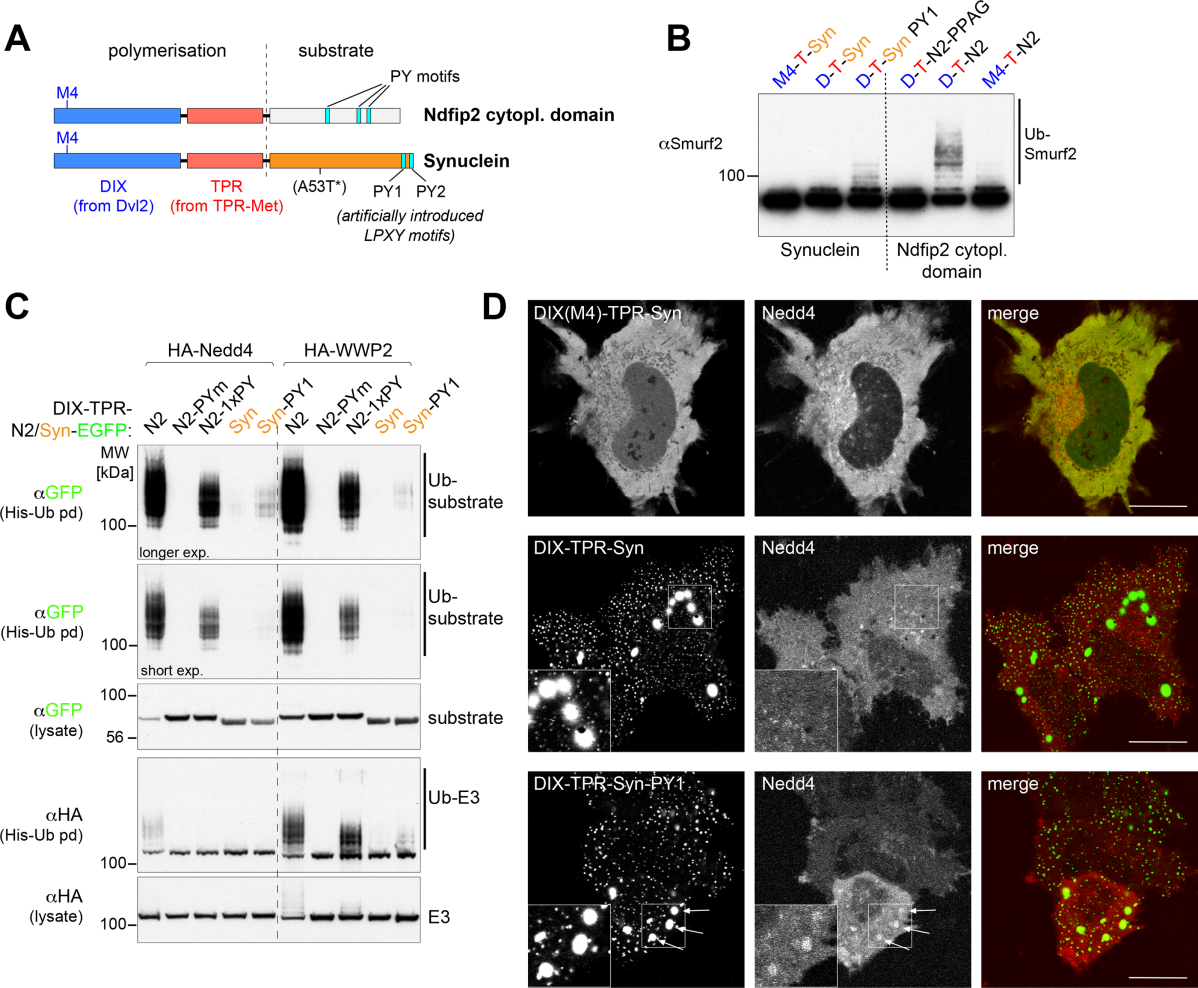
Clustering of alpha-synuclein does not result in efficient ubiquitination. (A) Schematic of the polymerization domains (DIX and TPR) fused to a soluble substrate, either the cytoplasmic domain of Ndfip2 containing three PY motifs or full-length alpha-synuclein with mutants that were used in this study. The location of the A53T mutation is indicated, but mutant protein was not used in Fig 4 (see Fig 5A). (B) *In vitro* ubiquitination assays using Smurf2 with purified recombinant proteins as indicated above the panels, showing efficient Smurf2 autoubiquitination when incubated with polymerization-competent DIX-TPR-Ndfip2 with intact PY motifs (D-T-N2), but not when all three PY motifs were mutated (D-T-PPAG). No Smurf2 autoubiquitination was observed with DIX-TPR-alpha-synuclein (D-T-Syn) and only modest effects were observed with an introduced weak PY motif (D-T-Syn PY1). (C) Western blots of His pull downs from HEK293T cells co-transfected with His-Ub, HA-tagged Nedd4, WWP2 and the GFP-tagged highly efficient polymerizing DIX-TPR fusions as indicated. N2-Pym, Ndfip2 with all three PY motifs mutated; N2-1xPY, Ndfip2 with one remaining LPxY motif. (D) Live-cell images showing single confocal sections of representative HeLa cells transfected with DIX-TPR-alpha-synuclein-EGFP either with polymerization defective M4 mutant (top left), wild-type alpha-synuclein (middle left) or introduced weak PY motif (bottom left panel) and co-transfected with mCherry-Nedd4 (center panels). Recruitment of Nedd4 into larger punctate aggregates was observed only in the presence of the weak PY motif (see arrows). Insets show enlarged versions of the outlined areas.

Clustering can be achieved using constructs that contain a DIX domain (from the Dishevelled protein Dvl2) and a dimerization domain from TPR-met (Fig 4A) [20]. Addition of sequences containing a PY motif (from the Ndfip2 protein) to this construct results in activation of Smurf2 *in vitro*, as shown by increased autoubiquitination (Fig 4B) [20], whereas a control containing a point mutation in the DIX domain (M4) that abolishes polymerisation does not. Despite the fact that alpha-synuclein filaments can activate Smurf2 (Fig 3C), a polymerisation construct bearing the full-length alpha-synuclein sequence did not do so (Fig 4B). Modifying the alpha-synuclein sequence to create a weak PY motif (by changing residues 116–119 from MPVD to LPTY) did allow some activation (Fig 4B), showing that the alpha-synuclein sequence was accessible and that even weak binding sites could be detected. The inactivity of wild-type alpha-synuclein in this assay suggests that activation by filaments cannot be mimicked by the mere clustering of monomers.

To extend these findings, we co-expressed the polymerising constructs (including GFP) with Nedd4 or WWP2 in HEK293 cells. Fig 4C shows that constructs bearing the Ndfip2 PY motifs were efficiently ubiquitinated by both Nedd4 and WWP2; this was abolished if the PY motifs were mutated, and was reduced if a single weak LPxY motif was present. Activation, as measured by autoubiquitination, of WWP2 and, to a lesser extent Nedd4, was also induced by these constructs (Fig 4C). In contrast, constructs bearing alpha-synuclein were only very weakly ubiquitinated by Nedd4, though addition of LPTY allowed some activity, and also led to detectable activation of WWP2. Microscopy confirmed that the alpha-synuclein constructs formed prominent clusters (whereas the non-polymerising M4 mutant did not), but that even large clusters did not attract co-expressed Nedd4 unless a PY motif was added (Fig 4D). Examination of fields of transfected cells showed that 0% contained Nedd4-positive clusters when the PY motif was absent, whereas with the LPTY sequence >50% of the transfected cells contained at least one Nedd4-positive cluster.

**Fig 5:**
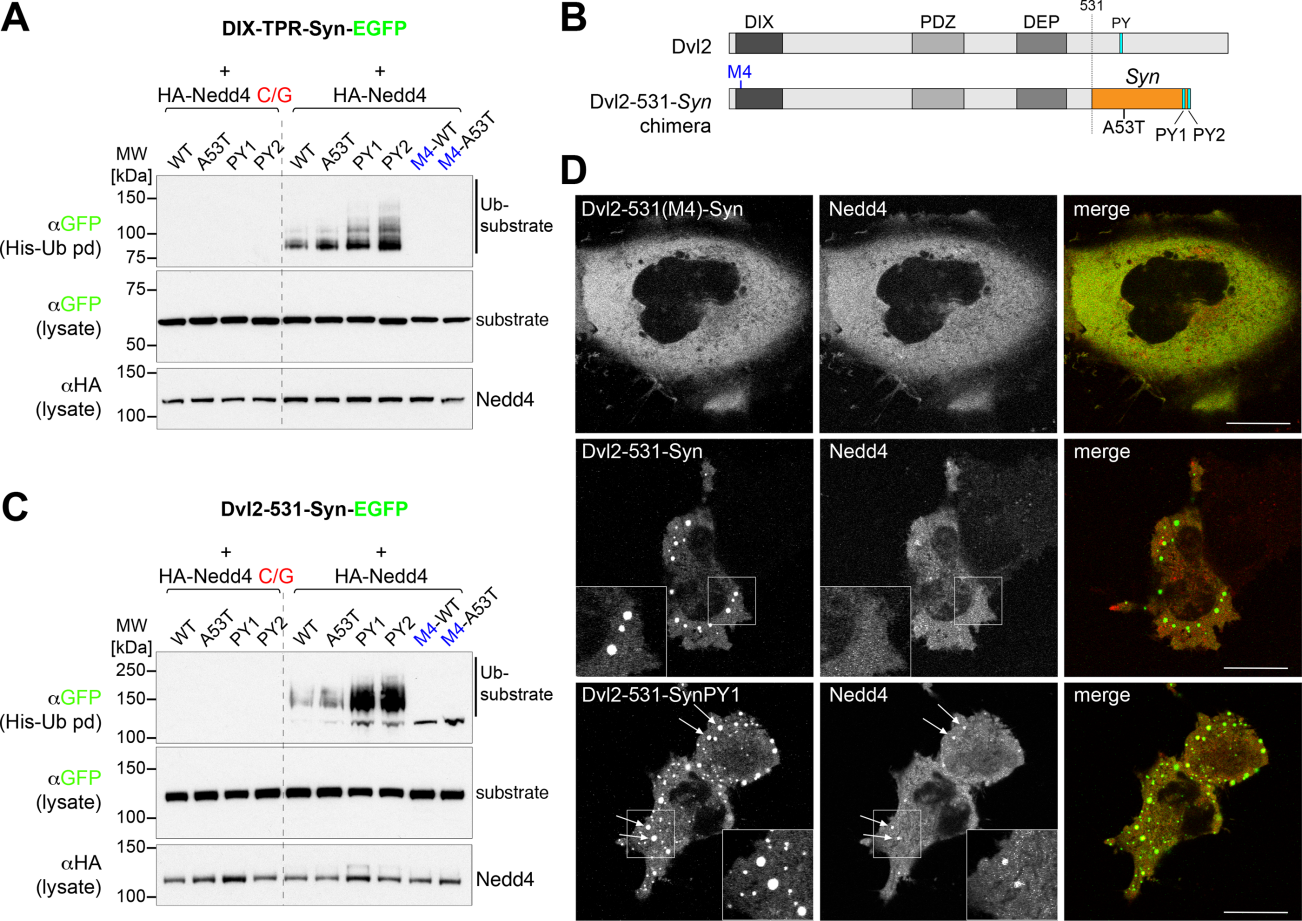
No difference in ubiquitination between clustered wild-type and A53T alpha-synuclein. (A,C) Western blots of His pull downs from HEK293T cells co-transfected with His-Ub, HA-tagged catalytically dead (C/G) or wt Nedd4 and the GFP-tagged polymerizing DIX-TPR fusions (A) or Dvl2-synuclein fusions (C) and their mutant versions as indicated. Clustered constructs with artificially introduced weak PY motifs are more efficiently ubiquitinated. (B) Schematic of the Dvl2-alpha-synuclein fusions used in (C). (D) Live-cell images showing single confocal sections of representative HeLa cells transfected with Dvl2-alpha-synuclein-EGFP either with polymerization defective M4 mutant (top left), wild-type alpha-synuclein (middle left) or introduced weak PY motif (bottom left panel) and co-transfected with mCherry-Nedd4 (center panels). As observed with the DIX-TPR-synuclein fusions (see Fig 4D) recruitment of Nedd4 into the larger punctate aggregates was observed only in the presence of the weak PY motif (see arrows). Insets show enlarged versions of the outlined areas.

Further evidence that clustered monomers are not equivalent to filaments is provided by analysis of the weak Nedd4-dependent ubiquitination that is detectable for the clustered proteins using highly sensitive detection methods (Fig 5A). Without PY motifs the ubiquitination is weak, but dependent on active Nedd4 and enhanced by clustering. Tellingly, however, modification of wild-type and A53T alpha-synuclein proteins was identical. This is in stark contrast to the *in vitro* ubiquitination of filaments, which was greatly reduced by the A53T mutation (Fig 1D), a result observed in multiple experiments with different batches of filamentous protein.

Similar results were obtained with a different construct in which full- length alpha-synuclein replaced the PY element at the C terminus of the complete dishevelled protein Dvl2 (Fig 5B), which polymerises particularly well [21]. Again, without the introduction of a PY motif, polymerised alpha-synuclein was only weakly ubiquitinated by Nedd4 (Fig 5C), and did not recruit Nedd4 (Fig 5D; 0% of the transfected cells showed co-localization of Nedd4 with the construct lacking PY, whereas with the PY motif >50% of the transfected cells contained Nedd4-positive structures). Thus, clustering of alpha-synuclein monomers is not sufficient to activate ubiquitin ligases, or to bind them, or to create a well-recognised substrate for ubiquitination.

Together, these findings strongly suggest that the formation of beta-sheet filaments creates a specific recognition site for Nedd4, thus allowing these filaments to be selectively and efficiently modified in cells compared to monomers.

## Discussion

We examined the previously reported ubiquitination of alpha-synuclein by members of the Nedd4 family of HECT ubiquitin ligases [4]. In particular, we sought to determine what feature of alpha-synuclein makes it a substrate for these enzymes. Our key finding is that beta-sheet-containing filaments, generated *in vitro* to mimic the pathological state of the protein, are excellent substrates for Nedd4 and, to a lesser extent, several other members of the Nedd4 family. In contrast, monomeric alpha-synuclein is a very poor substrate, as are clusters of protein created by fusion to a polymerising construct. *In vivo*, alpha-synuclein is thought to interact with membranes [1], a feature that can also favour recognition by ubiquitin ligases [20]; however, we have previously shown that clustering can substitute for membrane binding [20], suggesting that the key feature for ubiquitination is the filament state of alpha-synuclein.

We have also shown that recognition of alpha-synuclein filaments is not due to the presence of even weak PY-like motifs that can interact with the WW domains of Nedd4 ligases. Introduction of a PY-like motif makes alpha-synuclein behave more like a conventional substrate, but without this, recognition seems instead to be mediated by other interactions that require the C2 domain (e.g. in Smurf2), the HECT domain (e.g. in Itch) or both (e.g. in Nedd4). These interactions are evidently sufficient to overcome the normal auto-inhibition shown by these enzymes, most clearly in the case of Smurf2, presumably by competing with the normal inhibitory intramolecular interactions.

Together, these findings strongly suggest that the formation of beta-sheet-containing filaments creates a surface that is recognised by the ubiquitin ligases. Strikingly, A53T alpha-synuclein shows greatly reduced susceptibility to ubiquitination *in vitro*. The A53 residue lies close to a region that is thought to form the core of the beta-sheet-rich region [22]. Its mutation does not hinder filament formation, but could reduce the surface hydrophobicity of the beta-sheet, and thus reduce binding of the ubiquitin ligase. An alternative explanation could be that the mutation induces a subtle change in overall structure, but until the precise structures of these filaments are known, it is not possible to distinguish between these alternatives. We note that the weak ubiquitination of unassembled alpha-synuclein is not affected by the A53T mutation, consistent with it being relevant only in the context of the beta sheet structure.

The ability of a single point mutation to affect ubiquitination, together with the observation that the requirements for filament recognition differ between Nedd4 family ligases, indicates that the filament interactions are quite specific. Indeed, a previous study of *in vitro* ubiquitination using a reticulocyte lysate fraction as the source of ubiquitin ligase activity showed less efficient ubiquitination of filaments than of monomers [23]. This suggests that the filaments are not simply “sticky” or generically recognised as being unfolded. Their specific recognition by Nedd4 may be beneficial, and thus subject to evolutionary preservation.

Individuals with the dominantly inherited A53T alpha-synuclein mutation develop early-onset Parkinson’s disease [11], possibly because the mutant protein forms filaments more rapidly than its wild-type counterpart [13]. A plausible additional factor may be its relative lack of ubiquitination. We suggest that the recognition, ubiquitination and destruction of alpha-synuclein filaments, when they first form, helps to protect cells against the accumulation or spread of toxic forms of the protein, whilst the native protein remains unaffected. By inhibiting this process, A53T alpha-synuclein may favour more rapid generation of toxicity, and this may in turn contribute to the rapid development of Parkinson’ disease.

## Materials and methods

### Plasmids

The following eukaryotic expression plasmids have been described before: N-terminally HA- or mCherry-tagged NEDD4 family ligases (wt and catalytically-inactive mutants), His-Ubiquitin and DIX-TPR-Ndfip2 (wt and triple PPAG mutant) [16, 20, 21, 24]. For this study we also used a double PY motif mutant of DIX-TPR-Ndfip2 which left the third LPXY motif intact (1xPY). To polymerise full-length alpha-synuclein in cells we generated DIX-TPR-Synuclein (Fig 4) and alternatively fused synuclein to the first 531 amino acid residues of dishevelled, which replaced the natural PY motif of Dvl2 (Fig 5). Artificial weak PY motifs were introduced into the C-terminus of alpha-synuclein, PY1: aa116-119 (MPVD->LPTY), PY2: 137–140 (EPEA->LPSY). Additionally, the A53T mutant of alpha-synuclein was used. All *in vivo* polymerisation constructs were cloned into pEGFP-N1 (Clontech). For bacterial expression full length Nedd4-type ligases were cloned into pGEX6P-2 (GE Healthcare, Piscataway, NJ, USA). The following E3 deletions and mutants were used: Nedd4 ΔC2: aa141-900, Nedd4 HECT-only: aa521-900, Nedd4L HECT-only: aa576–955, Smurf2 ΔC2: aa141-748, Smurf2 HECT-only: aa371–748, Itch ΔC2: aa121-862, Itch HECT-only: aa486–862, ITCH WW domain 1–4 mutant (WWm): GW290,291VC (WW1), GW322,323VC (WW2), GW402,403VC (WW3), GW442,443VC (WW4) [16, 24]. All *in vitro* polymerisation constructs were cloned into a modified pET28a vector (Novagen) with a C-terminal triple HA tag, a PreScission cleavage site, glutathione s-transferase (GST) and a His tag. All constructs and point mutations were generated by standard procedures and verified by sequencing.

### Transfection and cell-based assays

Cell lines used in this study (2HEK93T and Hela) were acquired from the ATCC and were tested and confirmed negative for mycoplasma contamination. HEK293T cells were cultured and transfected essentially as described [16, 20, 21, 24]. Transient transfections of 293T cells were performed using Polyethylenimine (PEI MAX, linear, MW 40,000, Polysciences, Warrington, PA). Typically, for 2μg plasmid DNA, 5μl of a 1mg/ml solution of PEI were used and cells were harvested about 24 hours after transfection. His-ubiquitin pull downs were done under denaturing conditions. Typically, 1x10^6^ cells were harvested in 0.8ml 8M Urea 100mM Tris (pH7.4), 2mM NEM, 10mM Iodoacetamide, sonicated and incubated with 20μl His-tag Dynabeads (Life Technologies) at RT for several hours. The beads were washed three times in 8M Urea and eluted in sample buffer. Immunoblotting was done using standard procedures, and proteins were detected using antibodies from Sigma-Aldrich (anti-HA, H3663 mouse monoclonal), Roche (anti-GFP, 11814460001 mouse monoclonal), Abcam (Smurf2, ab53316 rabbit monoclonal), Millipore (anti-ubiquitin, 07–375 rabbit polyclonal) and BD Bioscience (anti-synuclein [Syn-1], 610786 mouse monclonal). For live-cell imaging HeLa cells were seeded into chambered coverglass (Lab-Tek) and transfected with either Polyethylenimine (PEI MAX, linear, MW 40,000, Polysciences, Warrington, PA) or Fugene HD transfection reagent (Promega).

### Recombinant protein expression and purification

All recombinant Nedd4-type E3 ligases and polymerisation constructs were expressed as GST-fusions. Plasmids were transformed into BL21 (DE3) CodonPlus-competent *Escherichia coli* (Stratagene) and expression was induced with 0.5mM isopropyl beta-D-thiogalactoside (IPTG) at 18°C overnight. Cell pellets were resuspended in PBS (0.5mM TCEP, 5% Glycerol) and lysed by sonication. GST-fusion proteins were affinity-purified from soluble bacterial lysates by using glutathione-sepharose (GE Healthcare) according to standard manufacturer's protocols and cleaved off GST while bound to the beads by a two hour incubation at 30°C with PreScission protease (GE Healthcare). Soluble recombinant proteins were either concentrated with Amicon Ultra centrifugation filter devices (Millipore) and stored as 5–10 mg/ml stocks at –80°C until required (Nedd4 ligases) or the concentration was adjusted to about 0.5-1mg/ml (polymerisation-competent proteins) and stored at –80°C.

### Filamentous assembly of alpha-synuclein

Bacterial expression and purification of alpha-synuclein proteins was done as described [25]. For filamentous assembly, purified alpha-synuclein proteins were incubated for up to 48 h at 450 rpm at 37°C in a shaking bacterial incubator [12]. The kinetics of filament formation were analysed by thioflavin T binding. Duplicate samples were taken at indicated times and 5 μM of thioflavin T in distilled water was added. Thioflavin fluorescence (induced by binding to beta sheet structures) was measured using excitation at 435nm, emission at 485 nm in a TECAN infinite 200Pro. Results shown in Fig 1B are the average of the duplicates. To quantitate filaments for use in ubiquitination assays, total protein concentration was determined in a Nanodrop spectrophotometer (ThermoFisher) (Extinction coefficient: 5960, Mw:14460 for WT and 14490 for A53T), then the assembled protein spun down at 45,000 rpm for 20 min, and the protein concentration of the supernatant determined and subtracted from the total protein amount.

### In vitro ubiquitination assays

*In vitro* ubiquitination experiments were set up essentially as described before [24 2018]. Assays (20 or 30μl) typically contained 50nM of human E1 (BostonBiochem, Cambridge, MA, USA), 400nM of the human E2 (UbcH5c, BostonBiochem), 10μg of ubiquitin (BostonBiochem), 100-250nM E3 and 200-600nM of synuclein or 600nM of DIX-TPR fusions (please see figure legends for experimental details). Assays were incubated at 30°C for 75–90 minutes and stopped by adding sample buffer. In the UbiCrest DUB assay deubiquitinases were added to the ubiquitination assay.
