# Correction: Ubiquitination of alpha-synuclein filaments by Nedd4 ligases

**DOI:** 10.1371/journal.pone.0202158

**Published:** 2018-08-07

**Authors:** The PLOS ONE Staff

## Notice of Republication

This article was republished on July 23, 2018 to correct errors to the ORCID iDs of the first and fourth authors introduced during the typesetting process. The publisher apologizes for the errors. Please download this article again to view the correct version.
